# One-pot synthesis of purple benzene-derived MnO_2_-carbon hybrids and synergistic enhancement for the removal of cationic dyes

**DOI:** 10.1038/s41598-018-22203-1

**Published:** 2018-03-12

**Authors:** Hyemin Kim, Nagahiro Saito

**Affiliations:** 1 Department of Materials Science and Engineering, Graduate School of Engineering, Nagoya University, Furo-cho, Chikusa-ku, Nagoya, 464-8603 Japan; 2 CREST-JST, Nagoya University, Furo-cho, Chikusa-ku, Nagoya, 464-8603 Japan

## Abstract

MnO_2_-carbon hybrid (MnO_2_-C-PBz) was simultaneously synthesized by a one-step solution plasma process (SPP) using a single precursor referred to as “purple benzene”, which was derived from the K^+^(dicyclohexano-18-crown-6 ether) complex. To clarify the synergistic effects on the cationic dye removal, MnO_2_-free carbon and carbon-free MnO_2_ samples were concurrently investigated. The results of adsorption for cationic dyes (methylene blue (MB) and rhodamine B (Rh B)) and anionic dye (methyl orange (MO)) revealed remarkably high affinity for cationic dyes. In particular, MnO_2_-C-PBz exhibited the highest adsorption capacity for MB, i.e., ~3 times greater than that of the others. In addition, MnO_2_-C-PBz exhibited a rapid, high decolorization ability at C_0_ = 10 mg L^−1^ (within a few seconds, ~99%) and at C_0_ = 100 mg L^−1^ (within 30 min, ~81%), and the theoretical maximum monolayer adsorption capacity was 357.14 mg g^−1^ as calculated from the Langmuir adsorption isotherm equation. Furthermore, compared with carbon-free MnO_2_, MnO_2_-C-PBz exhibited quite a good cyclic stability. We expect that our findings give rise to the understanding of the synergistic effects of MnO_2_-carbon hybrid, as well as role of each components for the cationic dye adsorption, and may open an innovative synthesis approach to inorganic-organic hybrid materials.

## Introduction

With the growth in the population and development of industries, there has been a considerable increase in the manufacture of food and products (including textiles, plastics, paper, and synthetic dyes), generating a large amount of effluent^1,2^. The treatment of wastewater containing toxic heavy metals (e.g., Hg, Cd, Pb, and As) and various organic substances (e.g., dyestuffs, pesticides, and hydrocarbons), which threaten the health of humans and animals, has generated considerable concern^3,4^. Among the different methods for the treatment of wastewater, adsorption has been attracting immense attention for its simplicity, cost-effectiveness, and reliability^3,5^. Hence, extensive studies have been focused on the search for efficient adsorbents.

Among the different adsorbents available, recently, manganese dioxide (MnO_2_) has been considered to be an effective adsorbent because of its environmental benignity, abundance, and cost-effectiveness^6,7^. In particular, MnO_2_ is highly suitable for the adsorption of cationic pollutants such as heavy metals and methylene blue (MB) because it exhibits a negatively charged surface over a wide range of pH (particularly high pH)^8,9^. However, metal nanoparticle based absorbent often limit a stability and reusability. The presence of various compounds in an aquatic environment, including ions, inorganic and organic pollutants, is known to lead to the self-aggregation of MnO_2_ nanoparticles, thereby reducing the available active sites^10–13^. To solve these issues, numerous studies have reported the formation of hybrid materials by the combination of various materials.

Recently, carbon materials (e.g., activated carbon (AC)^14,15^, carbon nanotubes (CNTs)^16^, graphene^17^, graphene oxide^18^, and mesoporous carbon^19^) have been combined with various inorganic compounds (e.g., mesoporous silica^15,20^, metal oxides^21^, and polymers^22^) to enhance the adsorption performance and stability because of their high surface area, good conductivity, and thermal and mechanical stabilities. Meanwhile, the typical method to prepare MnO_2_-carbon hybrid materials involves multi-step processes and a long processing time: (i) surface modification of carbon (e.g., CNTs and AC) by treatment in a strong acid, followed by (ii) deposition of MnO_2_ on the carbon surface by an aging process (e.g., refluxing, water-bath heating, and hydrothermal aging) for several hours under relatively high-temperature conditions^23–26^.

In our previous studies, nanostructured MnO_2_ has been successfully synthesized by the application of plasma, referred to as the “solution plasma process (SPP),” in a potassium permanganate aqueous solution without any additional chemical agents^27^ and with environmentally benign chemicals, i.e., sugar^28^. In addition, various carbon-based materials^29–31^ have been synthesized by a single-step SPP from organic solvents, such as benzene, toluene, or pyridine, under ambient temperature and pressure within several minutes.

In this study, based on this background, inorganic oxide-organic carbon hybrid materials (i.e., the MnO_2_-carbon hybrid) were synthesized by a single-step SPP. For the simultaneous synthesis of MnO_2_-carbon hybrids, benzene and KMnO were selected as the carbon and MnO_2_ precursors, respectively, and dicyclohexano-18-crown-6 ether (hereafter referred to as DCH18C6) was used as the main chemical for preparing the single precursor, referred to as “purple benzene.” DCH18C6 is used because of its high solubility in benzene, related to its hydrophilic (interior) and hydrophobic (exterior) properties; particularly, it exhibits a high affinity for potassium ions (K^+^) because of its size selectivity for guest cations^32–35^. Hence, MnO_4_^−^ is forcibly dissolved in benzene via the formation of a K^+^(DCH18C6) complex, affording “purple benzene” as the single precursor^36–38^.

The adsorption studies were evaluated with cationic dyes, i.e., MB and Rh B. To clarify the synergistic effects on the enhancement of the dye removal ability, of the MnO_2_-carbon hybrid, two types of MnO_2_-free carbon-based samples, synthesized from benzene and crown-ether-added benzene, respectively, were also investigated. In addition, the potential of the MnO_2_-carbon hybrid in comparison to carbon-free MnO_2_ as a reusable adsorbent was investigated by recyclability tests. Furthermore, to better understand adsorption phenomena, adsorption kinetics and isotherms were also examined.

## Results and Discussion

**Figure 1:**
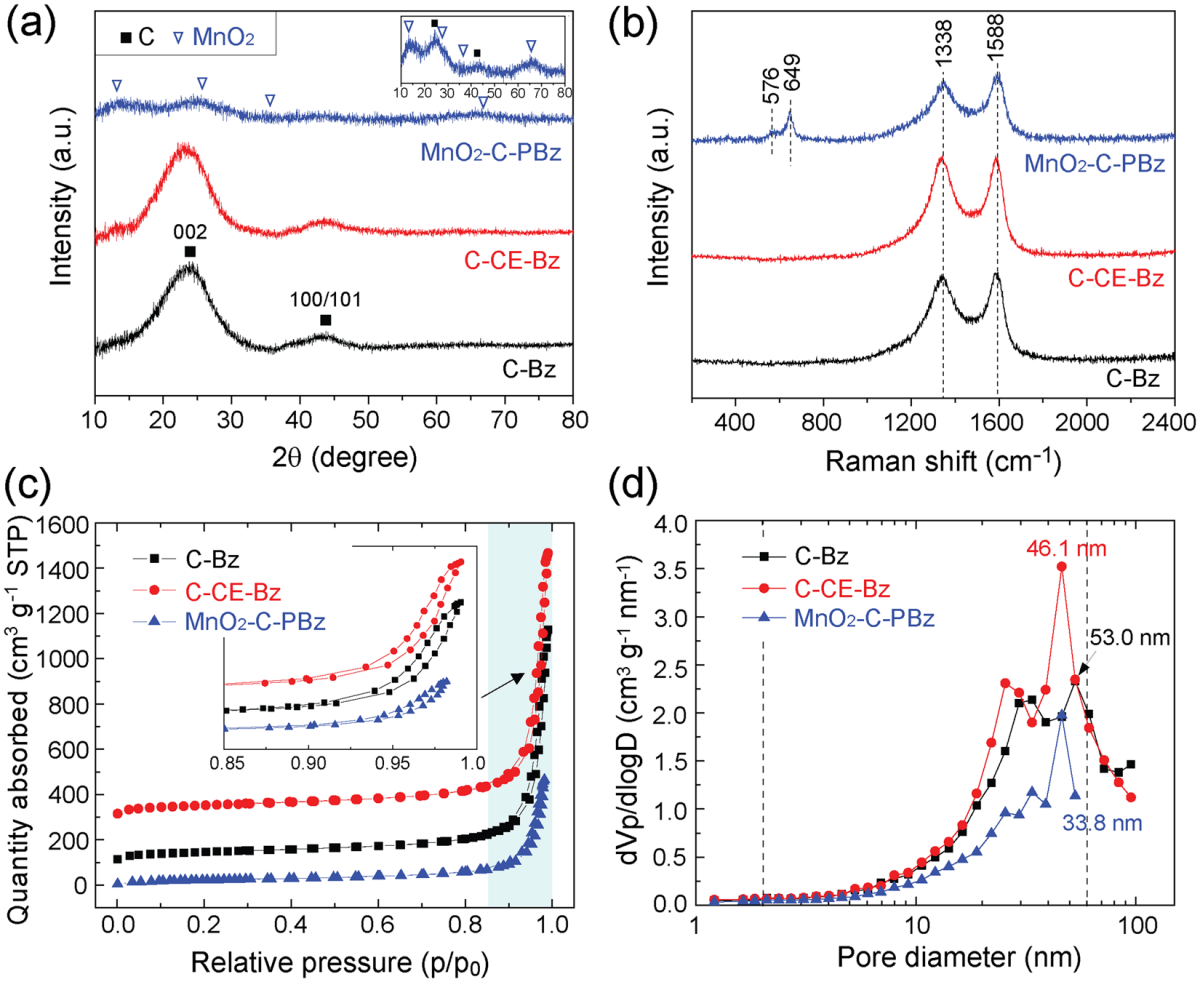
(**a**) XRD patterns, (**b**) Raman spectra, (**c**) N_2_ adsorption/desorption isotherms and hysteresis loop (inset), and (**d**) pore size distributions of C-Bz, C-CE-Bz, and MnO_2_-C-PBz.

The MnO_2_-carbon hybrid (MnO_2_-C-PBz) was successfully synthesized from purple benzene by SPP. To verify the effects of crown ethers or KMnO_4_, reference samples, i.e., C-Bz and C-CE-Bz, were also prepared as described in the experimental section. From the XRD data (Fig. 1(a)), broad peaks at 2θ = ~23.7° and ~43.7°, corresponding to the 002 and 100/101 planes of graphitic carbon, were observed for all samples^39^. C-Bz and C-CE-Bz exhibited almost the same XRD patterns, but the (002) peak of C-CE-Bz was slightly shifted toward a lower angle at 2θ = ~23.4°. Crown ethers and/or decomposed oxygen-containing by-products are considered to be embedded in the carbon matrix. In contrast, compared with C-Bz and C-CE-Bz, MnO_2_-C-PBz exhibited considerably weaker and broader peaks. In addition, new peaks at around 2θ = 12.9°, 25.8°, 35.4°, and 66.1° were observed for MnO_2_-C-PBz (inset: magnified XRD pattern). These peaks corresponded to potassium birnessite-type MnO_2_ (JCPDS# 80-1098)^40^. These results indicate the possibility that MnO_2_ is formed and distributed on the surface of the carbon matrix. This can be deduced from the XRD results of MnO_2_/C-Bz prepared by dispersing C-Bz in aqueous KMnO_4_ solution (see supplementary information; Fig. S1^†^). The formation of the MnO_2_-carbon hybrid was further confirmed by Raman spectroscopy. Two strong peaks corresponding to the D- and G-bands were observed at 1338 and 1588 cm^−1^, respectively, for all samples, indicating the presence of carbon material (Fig. 1(b))^41^. Clear peaks were observed at 576 and 645 cm^−1^, corresponding to the Mn-O stretching vibration in the MnO_6_ octahedral layer and the symmetric stretching vibration in the MnO_6_ octahedral framework, respectively^42^. These results confirmed the successful synthesis of the MnO_2_-carbon hybrid by a single-step SPP.

The surface area and pore structure of the adsorbents were characterized by N_2_ adsorption–desorption isotherm analysis. All samples exhibited type IV isotherms and a typical hysteresis loop of mesoporous materials (inset in Fig. 1(c))^43^. The specific surface area (SSA_BET_) values for C-Bz, C-CE-Bz, and MnO_2_-C-PBz were 148.67, 169.56, and 77.3 m^2^ g^−1^, respectively. With the addition of crown ether (i.e., C-CE-Bz), SSA_BET_ slightly increased, whereas with the addition of KMnO_4_ (i.e., MnO_2_-C-PBz), it considerably decreased. Figure 1(d) shows the pore size distributions. All samples exhibited a hierarchical porous structure with mesopores or macropores ranging between ~5 and 100 nm. The total pore volume and average pore size of C-Bz and C-CE-Bz were similar, whereas considerably lower values were observed for MnO_2_-C-PBz (Table 1). The effects of physical properties on adsorption capacity will be discussed in the section on adsorption studies.

**Table 1:** Physical properties of SPP-synthesized adsorbents, C-Bz, C-CE-Bz, and MnO_2_-C-PBz.

Samples	BET surface area (m^2^ g^−1^)	Total pore volume (cm^3^ g^−1^)	Average pore size (nm)
C-Bz	148.67	1.57	42.18
C-CE-Bz	169.56	1.78	42.04
MnO_2_-C-PBz	77.30	0.72	37.30

**Figure 2:**
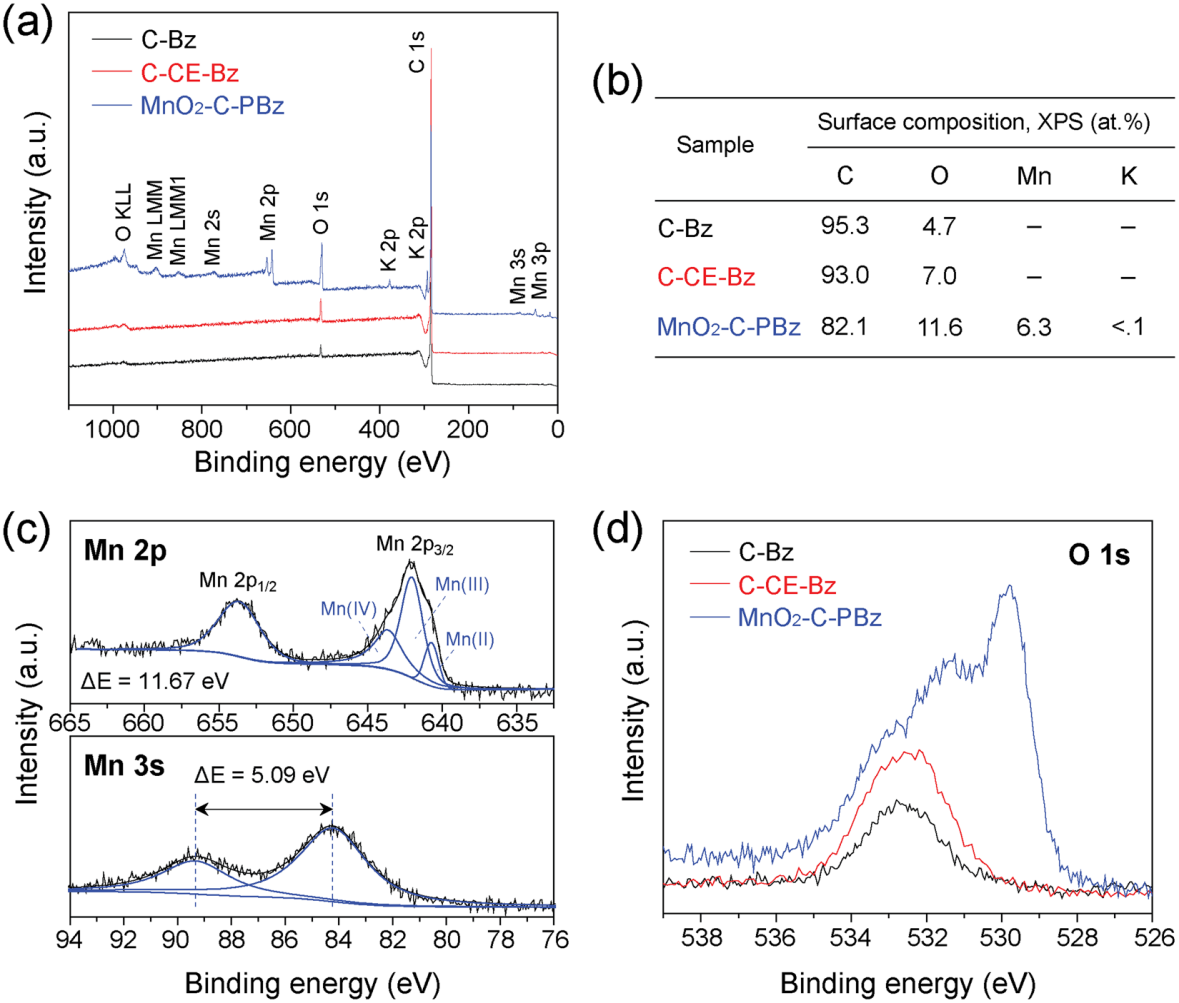
(**a**) XPS survey spectra and (**b**) XPS surface chemical composition of the as-prepared samples. High-resolution XPS spectra of (**c**) Mn 2p (upper side) and Mn 3 s (bottom side) of MnO_2_-C-PBz, and (**d**) O 1 s of all samples.

XPS analysis was carried out to investigate the surface elemental composition of the products and the oxidation state of manganese in MnO_2_-C-PBz. In comparison with MnO_2_-free samples (i.e., C-Bz and C-CE-Bz), Mn, O, and K were observed for MnO_2_-C-PBz (Fig. 2(a,b)), which clearly indicates that the MnO_2_-carbon hybrid is successfully synthesized by SPP. Figure 2(c) shows the high-resolution XPS spectra of Mn 2p and Mn 3 s. Two major peaks were observed at 653.74 and 642.07 eV, corresponding to the Mn 2p_1/2_ and Mn 2p_3/2_, respectively, and a peak separation (ΔE) value of 11.67 eV was in good agreement with literature values^44,45^. As shown in the bottom side of Fig. 2(c), the ΔE for Mn 3 s was 5.09 eV, indicative of a mixture of trivalent (Mn^3+^) and tetravalent (Mn^4+^) states^46,47^. This result is in agreement with the as described deconvolution peaks observed in the Mn 2p_3/2_ spectrum, which showed a mixture of Mn^4+^, Mn^3+^, and Mn^2+^. The O 1 s spectra (Fig. 2(d)) provided clear evidence for the formation of MnO_2_ because binding energy of metal oxide is different from those of the other oxygen species^48^. Compared with MnO_2_-free C-Bz and C-CE-Bz samples, MnO_2_-C-PBz exhibited new peaks at around 531–529 eV. Two strong peaks were observed at binding energy values of 529.8 and 531.3 eV, corresponding to oxygen species in the MnO_2_ lattice (O_latt._, Mn-O-Mn) and adsorbed oxygen species (O_ads._, Mn-OH), respectively^49^.

**Figure 3:**
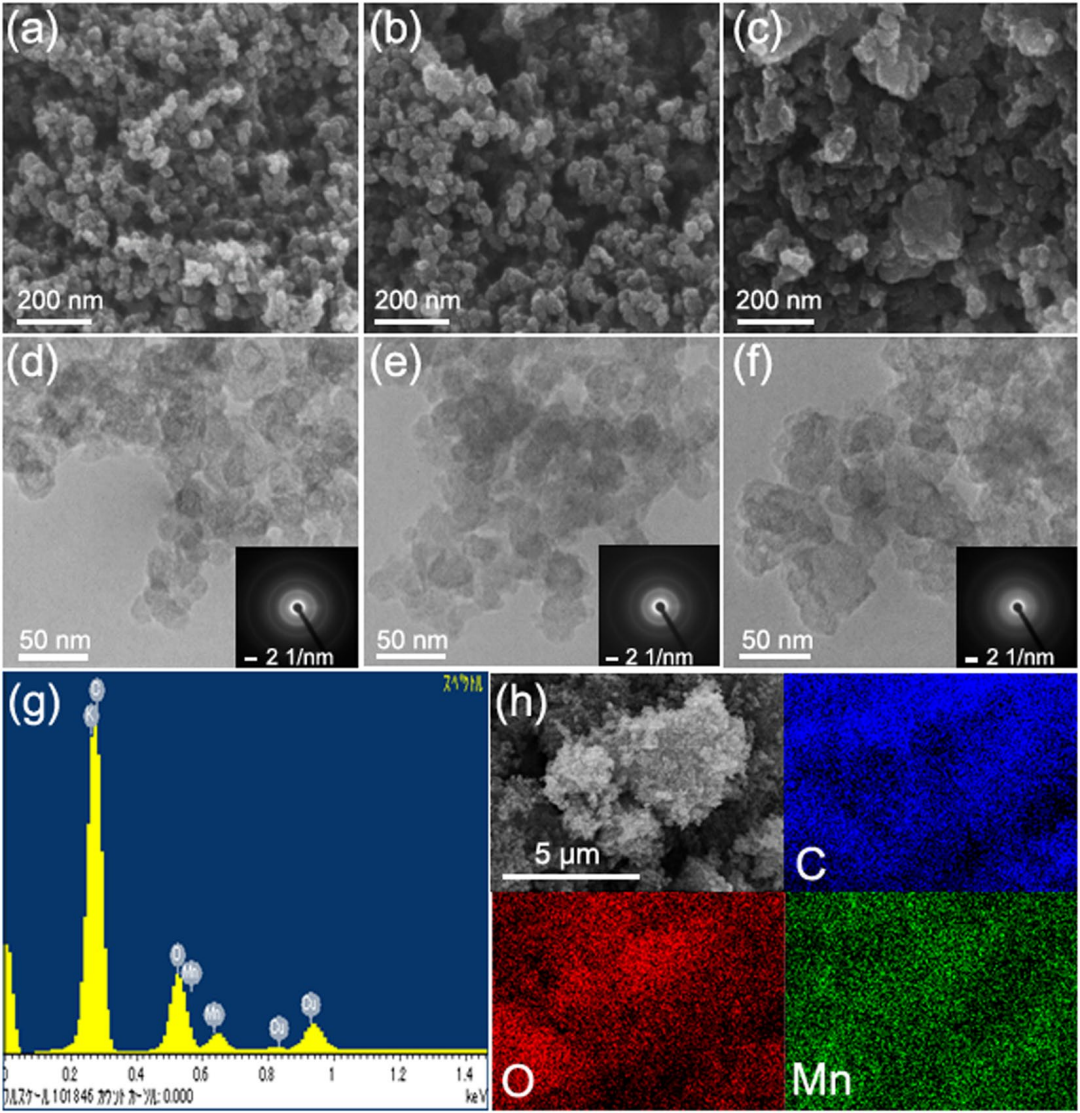
(**a**–**c**) FE-SEM images at 100 K magnification. (**d**–**f**) TEM images and SAED patterns (inset) of C-Bz (left), C-CE-Bz (middle), and MnO_2_-C-PBz (right). (**g**) EDX spectra and (**h**) elemental maps of C, O, and Mn of MnO_2_-C-PBz.

FE-SEM and TEM were used to examine C-Bz, C-CE-Bz, and MnO_2_-C-PBz. C-Bz exhibited relatively uniform spherical particles with a size of ~20 nm (Fig. 3(a,d)), and C-CE-Bz exhibited a slightly larger particle size of ~50 nm (Fig. 3(b,e)). On the contrary, MnO_2_-C-PBz exhibited non-uniform flat particles in a wide particle size range from tens to hundreds of nanometers (Fig. 3(c,f)). The selected-area electron diffraction (SAED) patterns of samples (Fig. 3(d–f) inset images) exhibited three broad diffuse rings, corresponding to the 002, 100/101, and 110 planes of carbon materials from the inside diffraction ring, respectively^50^. C-Bz and C-CE-Bz exhibited almost identical diffraction patterns, whereas MnO_2_-C-PBz showed a weaker, broader diffraction pattern without any distinct diffraction patterns of MnO_2_. This result indicated that amorphous MnO_2_ is formed and loaded on the carbon surface, as evidenced from the weaker, broader SAED pattern compared with those of C-Bz and C-CE-Bz as well as the XRD results. In addition, the EDS spectrum (Fig. 3(g)) and elemental mapping results (Fig. 3(h)) clearly indicated that C, O, and Mn are present and that Mn and O are uniformly dispersed in MnO_2_-C-PBz.

**Figure 4:**
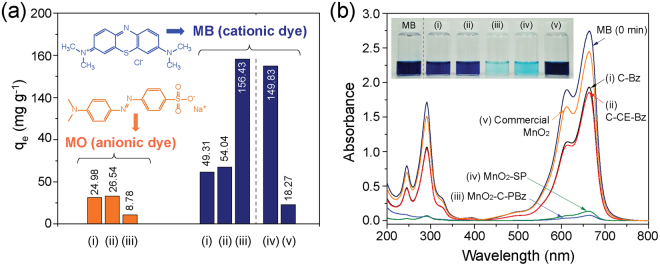
(**a**) Comparison of the equilibrium adsorption capacities of (i) C-Bz, (ii) C-CE-Bz, (iii) MnO_2_-C-PBz, (iv) MnO_2_-SP, and (v) commercial MnO_2_ for the anionic dye (MO) and the cationic dye (MB). (**b**) UV-visible spectra and the MB dye solution after adsorption using each sample (inset images) (297 K, 0.6 g L^−1^ of adsorbent, and natural pH (6.5 ± 0.5)).

To observe the adsorption of MO and MB (pH = 6.5 ± 0.5) on the as-prepared samples, adsorption tests were carried out in MO and MB stock solutions. The adsorption behavior of MO and MB was clearly different (Fig. 4(a)). All samples exhibited a higher adsorption capacity for MB than MO, indicating that the as-synthesized samples are suitable for the adsorption of cationic dye molecules. Notably, (i) C-Bz and (ii) C-CE-Bz exhibited higher adsorption capacities than (iii) MnO_2_-C-PBz in an MO solution, whereas the opposite tendency was observed in an MB solution. In particular, despite the fact that the SSA_BET_ values of C-Bz and C-CE-Bz were greater than that of MnO_2_-C-PBz, MnO_2_-C-PBz exhibited around three times higher adsorption capacity than the other samples, i.e., (i) C-Bz, 49.31 mg g^−1^, (ii) C-CE-Bz, 54.04 mg g^−1^, and (iii) MnO_2_-C-PBz, 156.43 mg g^−1^, under the same conditions for the MB solution. In addition, to investigate the effect of MnO_2_ in the hybrid products, MB adsorption experiments were carried out using (iv) MnO_2_-SP (synthesized by the same method, i.e., SPP^28^. see supplementary information; Table S1^†^) and (v) commercial MnO_2_. Compared with (iv) MnO_2_-SP and (v) commercial MnO_2_, the MnO_2_-carbon hybrid exhibited higher adsorption capacity (Fig. 4(a)). The excellent adsorption ability of the MnO_2_-carbon hybrid was clearly demonstrated by UV-visible spectra and MB dye solution color (inset image in Fig. 4(b)) after equilibrium adsorption (Fig. 4(b)). Additionally, Rh B adsorption behavior of MnO_2_-carbon hybrid also exhibited good adsorption capacity and removal efficiency compared with anionic dye MO (see supplementary information; Fig. S2^†^). Therefore, these results suggest that the as-synthesized MnO_2_-carbon hybrid is suitable for the removal of cationic dyes; particularly, the adsorption ability is enhanced using a hybrid with carbon materials. The adsorption behavior and effects of MnO_2_ and carbon are investigated in detail in the following section on adsorption and isotherm studies.

**Figure 5:**
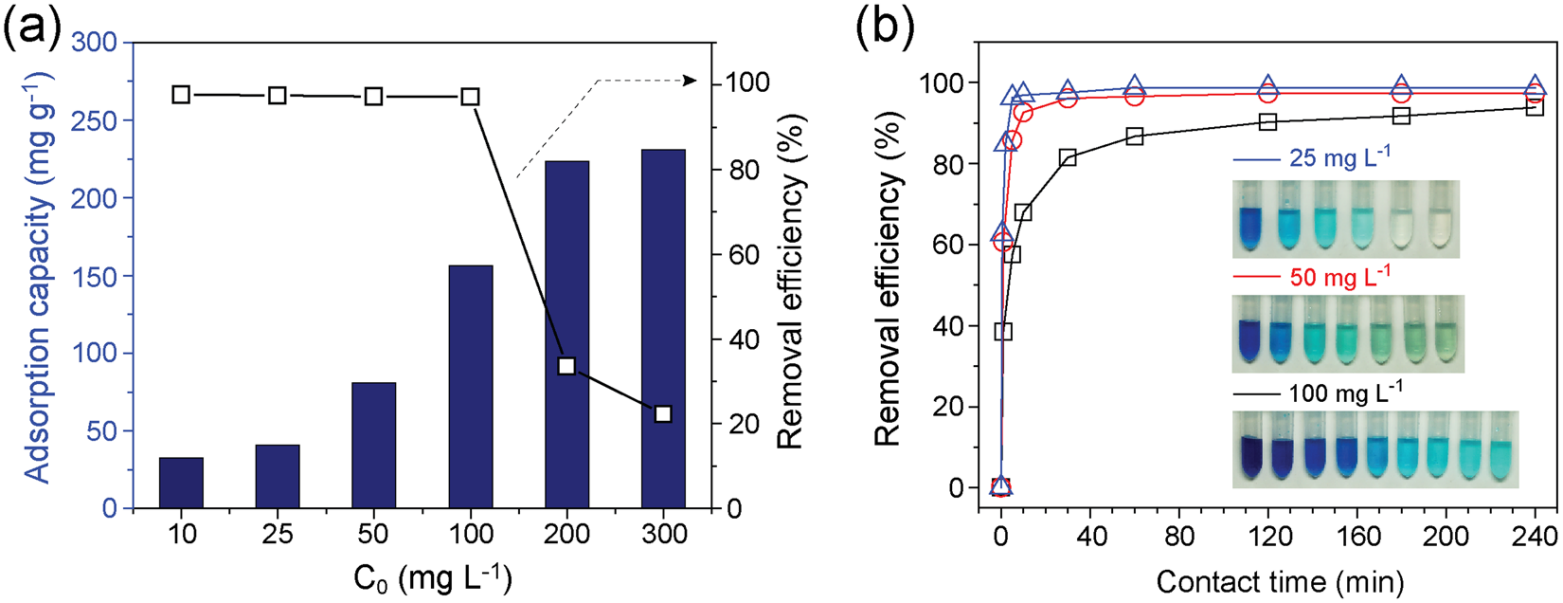
(**a**) Adsorption capacity (bar graph) and removal efficiency (–□–) of MnO_2_-C-PBz at different initial MB concentrations (C_0_ = 10, 25, 100, 200, and 300 mg L^−1^) at equilibrium. (**b**) Dependence of time on MB removal efficiency (C_0_ = 25, 50, and 100 mg L^−1^) and digital photographs (inset) showing the decolorization of MB by MnO_2_-C-PBz (297 K, 0.6 g L^−1^ of adsorbent, and natural pH (6.5 ± 0.5)).

Based on the above results, the adsorption ability of MnO_2_-C-PBz with the best adsorption characteristics was investigated in detail. The dependence of MnO_2_-C-PBz adsorption capacity on initial MB concentrations was evaluated to confirm the efficacy of the adsorbent. With the increase in initial MB concentrations from 10 to 300 mg L^−1^, the adsorption capacity of MnO_2_-C-PBz increased from 32.58 to 230.86 mg g^−1^, whereas the dye removal efficiency decreased (Fig. 5(a)). The removal efficiency was observed to be greater than ~93% up to C_0_ = 100 mg L^−1^, and it dramatically decreased to ~33.5% at C_0_ = 200 mg L^−1^ and to ~22.3% at C_0_ = 300 mg L^−1^. In addition, with the immediate addition of a significantly low initial concentration (C_0_ = 10 mg L^−1^) of MnO_2_-C-PBz, MB was rapidly removed within 10 s (~99%); hence, adsorption experiments are carried out at high concentrations of greater than 25 mg L^−1^ to confirm the dependence of time on adsorption behavior. Removal efficiencies of greater than ~97% and ~92% within 10 min were observed for C_0_ values of 25 and 50 mg L^−1^, respectively, and a removal efficiency of greater than ~81% at a C_0_ of 100 mg L^−1^ was observed within 30 min (Fig. 5(b)). Moreover, MB was effectively decolorized. As can be observed in the inset images of Fig. 5(b), the blue solution color clearly disappeared at a low initial MB concentration (25 mg L^−1^), indicative of the effective decolorization ability of MnO_2_-C-PBz. Although the blue color did not completely disappear at higher initial concentrations (i.e., 50 and 100 mg L^−1^), considerable decolorization was observed by comparison of the initial MB solution color. Thus far, the results indicate that MnO_2_-C-PBz possesses the highly fast and effective MB removal abilities.

**Figure 6:**
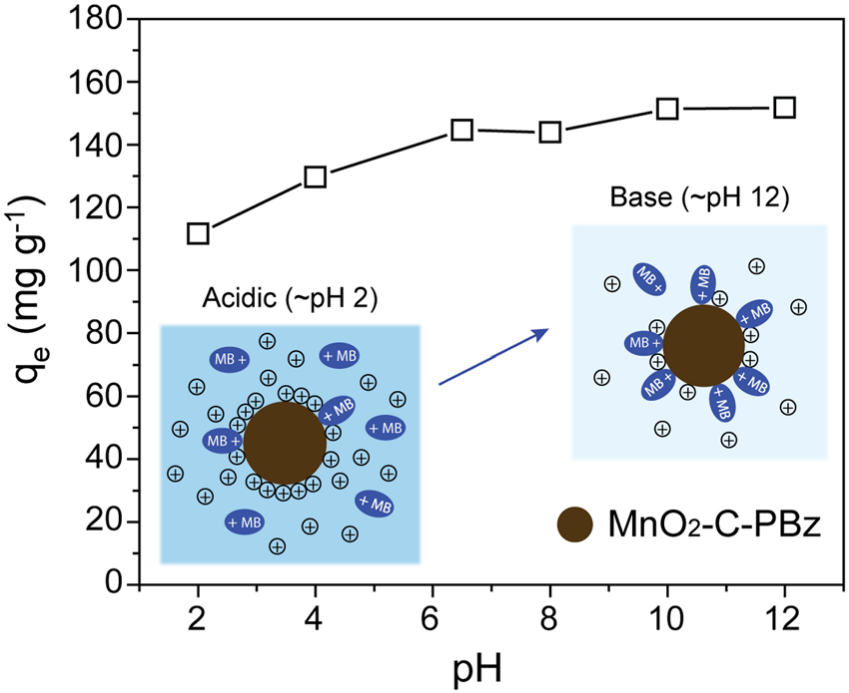
Adsorption capacity of MnO_2_-C-PBz at different pH values from 2 to 12 (297 K and 0.6 g L^−1^ of adsorbent).

The effects of pH were investigated because solution pH is an important factor affecting the adsorption of dye molecules in an aqueous system. With the increase in pH from 2 to 12, the adsorption capacity of MnO_2_-C-PBz for MB increased from around 111.6 to 151.8 mg g^−1^ (Fig. 6). *Murray*^8^ and *Fendorf et al*.^9^ independently investigated the pH of the zero point of charge of MnO_2_ (especially δ-phase MnO_2_), which was verified to be around ~2.4 and 2.7. These values indicated that MnO_2_ is negatively charged over a wide range of pH (pH > ~3). The MnO_2_-C-PBz surface apparently became more negatively charged with increasing solution pH.

The adsorption mechanism in hybrid nanocomposite system, various interactions between adsorbent and adsorbate might have possibility to contribute adsorption capability. The possible interactions between MnO_2_-carbon hybrid and MB molecules can be summarized as follows; (i) MnO_2_: the strong electrostatic forces of attractions between negatively charged MnO_2_ and positively charged MB^+^, (ii) π-π stacking interactions between bulk π-system of sp^2^-carbon and C=C or aromatic ring of MB molecules, and (iii) hydrogen bonding between oxygen-containing functional groups in carbon moieties derived from crown ether-containing precursor and MB molecules. Among these mechanism, we regard that electrostatic forces of attraction are main adsorption mechanism in our hybrid system based on the results of adsorption capability (Fig. 4) and pH-dependent of MB adsorption behavior (Fig. 6).

**Figure 7:**
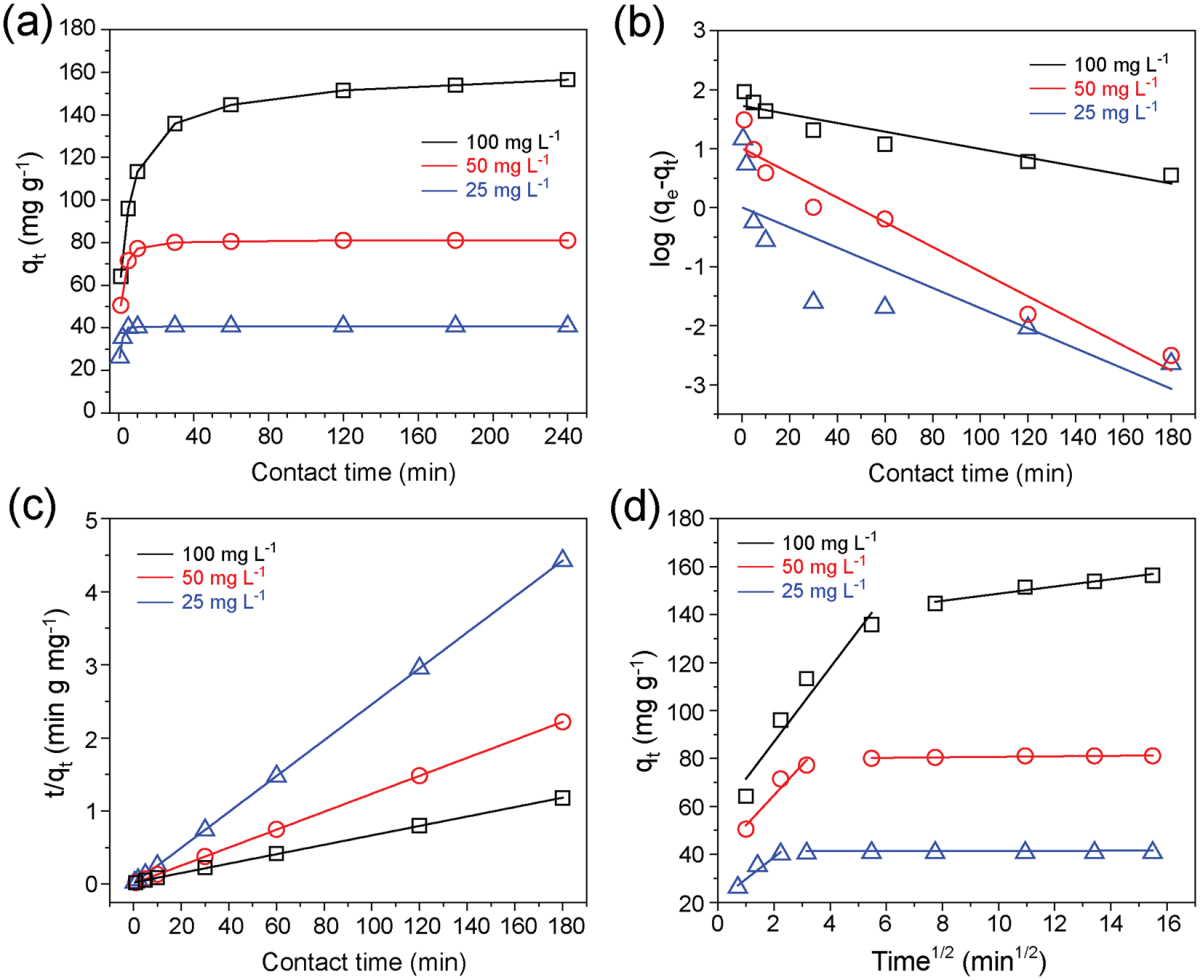
(**a**) Adsorption capacity as a function of contact time using the (**b**) pseudo-first-order, (**c**) pseudo-second-order, and (**d**) intraparticle diffusion models proposed by Weber and Morris for the adsorption of MB on MnO_2_-C-PBz at different initial concentrations (297 K, C_0_ = 25, 50, and 100 mg L^−1^, 0.6 g L^−1^ of adsorbent, and natural pH (6.5 ± 0.5)).

Three initial concentrations of dye solution, 25, 50, and 100 mg L^−1^, respectively, were selected to investigate the dependence of adsorption capacity on contact time. The equilibrium adsorption capacity increased with increase in the initial concentration (Fig. 7(a)). Rapid adsorption occurred at the initial stage (~10 min) and then attained equilibrium gradually. This result is related to the presence of a large number of active sites at the start of adsorption, followed by the decrease in the number of available active sites. To better understand the adsorption of MB on MnO_2_-C-PBz, adsorption kinetics was further examined. The obtained experimental kinetic data were fitted by the linear forms of two kinetic models: the pseudo-first-order (PFO, eq. (1)) and pseudo-second-order (PSO, eq. (2)) models, respectively^51,52^. These two linear forms can be described by eqs (1) and (2), respectively:

1 $${\rm{l}}{\rm{o}}{\rm{g}}({q}_{e}-{q}_{t})=\,{\rm{l}}{\rm{o}}{\rm{g}}({q}_{e})-\frac{{k}_{1}t}{2.303}$$

2 $$\frac{t}{q}=\frac{1}{{k}_{2}{q}_{e}^{2}}+\frac{1}{{q}_{e}}t$$

where *q*_*e*_ and *q*_*t*_ represent the adsorption capacities (mg g^−1^) at equilibrium and contact time (t, min), respectively, and *k*_1_ (min^−1^) and *k*_2_ (g mg^−1^ min^−1^) are the equilibrium rate constants for the PFO and PSO models, respectively. *k*_1_ was determined from the slope of log(*q*_*e*_ − *q*_*t*_) vs. *t* (Fig. 7(b)) obtained from the linear plots fitted using eq. (1), and *k*_2_ and *q*_*e*_ were determined from the slope and intercept of *t*/*q*_*t*_ vs. *t* (see Fig. 7(c)) obtained from the linear plots fitted using eq. (2). In addition, the initial (*t* → 0) adsorption rate *h* (mg g^−1^ min^−1^) was calculated by eq. (3)^51^:

3 $$h={k}_{2}{q}_{e}^{2}$$

As summarized in Table 2, the correlation coefficient (*R*^2^) values clearly indicated that the adsorption of MB on MnO_2_-C-PBz follows the PSO model (*R*^2^ > 0.9997) compared with the PFO model (*R*^2^ > 0.6938). The calculated values for the equilibrium adsorption capacity (*q*_*e*,*cal*_) from the PSO model were 40.65, 81.30, and 156.25 mg g^−1^ at C_0_ = 25, 50, and 100 mg L^−1^, respectively. These values are almost same as those obtained from experiments (*q*_*e,exp*_). This result indicates that the adsorption of MnO_2_-C-PBz follows the PSO model, with the rate-limiting step involving chemisorption between the adsorbate and adsorbent^52^. Therefore, the adsorption capacity of MnO_2_-C-PBz is considered to be greater than those of the other reference samples (i.e., C-Bz and C-CE-Bz) despite its lower surface area (C-Bz: 148.67 m^2^ g^−1^; C-CE-Bz: 169.56 m^2^ g^−1^; MnO_2_-C-PBz: 77.3 m^2^ g^−1^). In addition, the *k*_2_ and *h* values decreased with the increase in initial MB concentrations, indicating that adsorption reaches a plateau more rapidly at a low initial concentration^53^. However, it is difficult to determine the diffusion mechanism using only two previous kinetic models; hence, the linear form of the intraparticle diffusion kinetic model (proposed by Weber and Morris)^54^ was also applied at different initial MB concentrations to further investigate sorption using the following equation:

4 $${q}_{t}={k}_{ip}{t}^{1/2}+C$$

**Table 2:** Parameters of the PFO and PSO kinetic models for the adsorption of MB on MnO_2_-C-PBz.

Experimental data		PFO model			PSO model			
C_0_ (mg L^−1^)	*q*_*e*_,_*exp*_ (mg g^−1^)	*q*_*e*,*cal*_ (mg g^−1^)	*k*_1_ (min^−1^)	*R* ^2^	*q*_*e*,*cal*_ (mg g^−1^)	*k*_2_ (g mg^−1^ min^−1^)	*h* (g mg^−1^ min^−1^)	*R* ^2^
25	40.63	1.01	0.03938	0.6938	40.65	0.15925	263.1483	0.9999
50	81.07	10.15	0.04813	0.9532	81.30	0.02073	137.0189	0.9999
100	156.43	53.04	0.00299	0.8945	156.25	0.00186	45.4106	0.9997

The data obtained from eq. (4) exhibited two linear portions (Fig. 7(d)), indicating that adsorption can be described by a two-stage process: (i) the external surface adsorption stage, i.e., mass transfer through boundary-layer diffusion, and (ii) the gradual sorption stage, i.e., intraparticle diffusion^55^. *k*_*ip*_ represents the adsorption rate (mg g^−1^ min^−1/2^), and *C* (mg g^−1^) is the value related to boundary thickness. Steep slopes were observed at the initial portion, and plateau slopes were observed at the second stage (Fig. 7(d)). Initially, rapid adsorption was observed, which slowly attained equilibrium. This result suggests that the adsorption of MB on MnO_2_-C-PBz is controlled by external surface adsorption, followed by intraparticle diffusion^56^. In addition, the rate constants (*k*_*ip*_) increased with the increase in initial MB concentrations (*k*_*ip*_: 9.05 (at 25 mg L^−1^) < 12.60 (at 50 mg L^−1^) < 15.45 (at 100 mg L^−1^)) because a high initial MB concentration provides a high driving force to overcome the mass-transfer resistance of MB between the aqueous and solid phases, leading to a high rate for the adsorption of MB molecules on the MnO_2_-C-PBz adsorbent^57^.

The study of adsorption isotherms can provide insights into adsorption behavior, and efficient adsorbents can be designed on the basis of the obtained parameters. Hence, equilibrium adsorption data are analyzed using the linear form of the Langmuir adsorption isotherm model, which is based on the hypothesis of monolayer adsorption using a homogeneous adsorbent surface, and the Freundlich adsorption isotherm model, which is based on the hypothesis of multilayer adsorption on a heterogeneous adsorbent surface^58^. The linear form of the Langmuir adsorption isotherm model is described as follows:

5 $$\frac{{C}_{e}}{{q}_{e}}=\frac{{C}_{e}}{{q}_{max}}+\,\frac{1}{b{q}_{e}}$$

where *C*_*e*_, *q*_*e*_, and *q*_*max*_ represent the concentrations of the dye solution at equilibrium (mg L^−1^), adsorption capacity at equilibrium (mg g^−1^), and the theoretical maximum monolayer adsorption capacity of the adsorbent (mg g^−1^), respectively, and *b* is the Langmuir constant (L mg^−1^). The *q*_*max*_ and *b* values were determined from the slope and intercept of the linear fitted plot of *C*_*e*_/*q*_*e*_ vs. *C*_*e*_ (Fig. S3(a)^†^). Furthermore, the separation factor *R*_*L*_ (also known as the equilibrium parameter), providing information about the favorability of adsorption in the adsorbate/adsorbent system, was calculated using eq. (6)^59^:

6 $${R}_{L}=\frac{1}{1\,+\,b{C}_{0}}$$

*R*_*L*_ represents the shape of the isotherm: (i) *R*_*L*_ > 1 (unfavorable), (ii) *R*_*L*_ = 1 (linear), (iii) 0 < *R*_*L*_ < 1 (favorable), and (iv) *R*_*L*_ = 0 (irreversible)^59,60^.

The linear form of the Freundlich isotherm model is described as follows^61^:

7 $${\rm{l}}{\rm{n}}({q}_{e})=\,{\rm{l}}{\rm{n}}({K}_{F})+\,\frac{1}{n}\,{\rm{l}}{\rm{n}}({C}_{e})$$

where *K*_*F*_ is the Freundlich constant (mg g^−1^), related to the adsorption capacity, and *1*/*n* is the heterogeneity parameter: (i) *1*/*n* < 1 (favorable, heterogeneous adsorption) and (ii) *1*/*n* > 1 (unfavorable)^62^. These factors can be used to describe the multilayer adsorption on a heterogeneous adsorbent surface^58,62^.

Table 3 shows the calculated isotherm parameters from the linear forms of the Langmuir and Freundlich adsorption models and fitted lines presents in Fig. S3(b)^†^. The high *R*^2^ values indicate that the two models successfully explain the favorable adsorption of MB on MnO_2_-C-PBz. The theoretical maximum monolayer adsorption capacity (*q*_*max*_) was 357.14 mg g^−1^ as calculated from the Langmuir adsorption isotherm model. Compared with various adsorbents, including carbon, metal oxide, and metal-oxide-carbon hybrid adsorbents reported in literatures as shown in Table S2, MnO_2_-C-PBz exhibited a significantly high adsorption capacity. In particular, our MnO_2_-C-PBz showed very high adsorption capacity for MB compared with other similar metal oxide-carbon hybrid adsorbents, suggesting that it could be a promising adsorbent for application of cationic pollutants removal. The *R*_*L*_ values (0.00811–0.19693) in the range of 0–1 (Table 3) indicated the favorable adsorption of MB on MnO_2_-C-PBz. In addition, a higher correlation coefficient for the Freundlich model (*R*^2^ = 0.9607 > 0.9104) indicated that the adsorption of MB on MnO_2_-C-PBz is not restricted only to monolayer adsorption^62^. A *1*/*n* value of 0.6516 reflected that the adsorption of MB is favorable on the heterogeneous sites of MnO_2_-C-PBz.

**Table 3:** Parameters for the Langmuir and Freundlich adsorption isotherm models for the adsorption of MB on MnO_2_-C-PBz.

Langmuir adsorption isotherm model		Freundlich adsorption isotherm model	
*q*_*max,cal*_ (mg g^−1^)	357.14	*1*/*n*	0.6516
*b* (L mg^−1^)	0.4078	*K*_*F*_	6.4353
*R*_*L*_	0.00811–0.19693	*R* ^2^	0.9607
*R* ^*2*^	0.9104		

**Figure 8:**
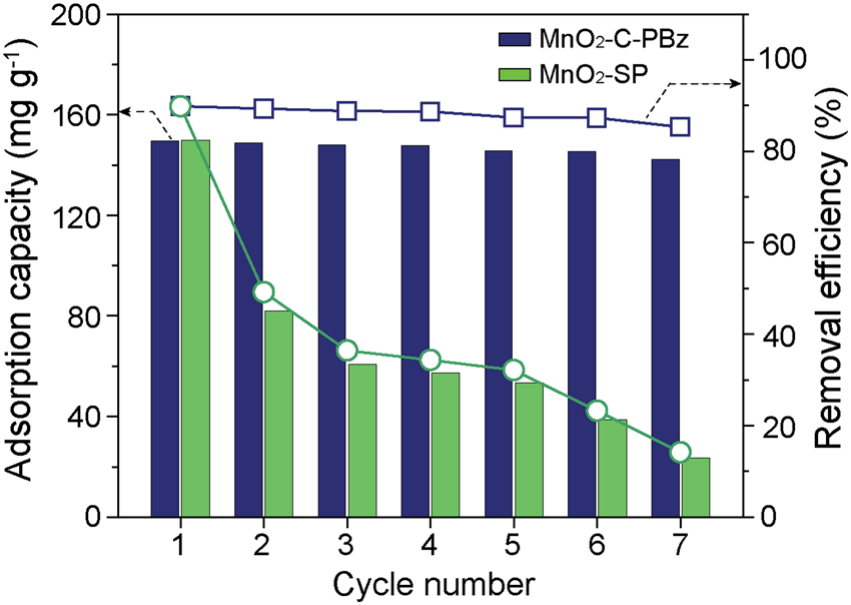
Reusability tests of MnO_2_-C-PBz and MnO_2_-SP for the removal of MB (bar graph = adsorption capacity (mg g^−1^), symbol-line graph = removal efficiency (%), C_0_ = 100 mg L^−1^, adsorbent dosage = 0.6 g L^−1^, and contact time = 180 min).

To confirm the reusability of the MnO_2_-carbon hybrid adsorbent, multi-recycle tests were performed and compared to the carbon-free MnO_2_-SP synthesized by SPP. As can be seen in Fig. 8, in the first cycle, MnO_2_-C-PBz and MnO_2_-SP exhibited similar adsorption capacities (~149.66 and ~149.83 mg g^−1^) and removal efficiencies (~89.78% and ~89.81%). In the second cycle, however, the removal efficiency of MnO_2_-SP dramatically decreased from 89.8% to 49.2%, whereas that of MnO_2_-C-PBz slightly decreased to around 0.49%. As the cycle repeats, the removal efficiency of MnO_2_-SP gradually decreased, but that of MnO_2_-C-PBz remained stable. Consequently, after seven cycles, the MnO_2_-C-PBz still maintained considerable dye removal performance and stability as compared with those of MnO_2_-SP. Although large SSA of MnO_2_-SP provides high adsorption capacity and removal efficiency in the first cycle, MB molecules could be blocked in the micropores or small mesopores; therefore, the efficient desorption of MB molecules from the adsorbent does not occur. Meanwhile, MnO_2_-C-PBz shows no obvious degrading after cycle tests (see Fig. S4^†^), which demonstrates immense potential for reusable adsorbent. From the results obtained thus far, the synergistic effects related to the presence of MnO_2_ and the high stability of carbon for the removal of cationic dyes have been clearly confirmed.

## Conclusion

In this study, we first report a one-pot synthesis of MnO_2_-carbon hybrid (MnO_2_-C-PBz) by applying plasma in single-precursor “purple benzene” derived from K^+^(DCH18C6) complex formation, and its performance for the adsorption of cationic dye (i.e., MB) was investigated. The MnO_2_-C-PBz showed around 3 times greater adsorption capacity than those of MnO_2_-free carbon (C-Bz and CE-Bz), and exhibited quite a good cyclic stability than carbon-free MnO_2_ (MnO_2_-SP). In addition, the adsorption kinetics and isotherm studies demonstrated that MnO_2_-C-PBz is a heterogeneous adsorbent, which is accompanied by complex adsorption, with both chemisorption and physisorption. From the results obtained thus far, the MnO_2_-carbon hybrid is an efficient and reusable adsorbent for the removal of cationic dyes, which attributed to adsorption properties of both MnO_2_ and carbon. This finding will be useful for understanding the synergistic effects of the MnO_2_-carbon hybrid for the adsorption of cationic dye and contributing an innovative single-step synthetic approach for inorganic oxide-organic carbon hybrid materials.

## Methods

### Chemicals and materials

Benzene (C_6_H_6_, >99.5% purity, Kanto Chemical Co., Inc., Japan) and potassium permanganate (KMnO_4_, >99.3% purity, Wako Pure Chemical Industries Ltd., Japan) were selected as the carbon and manganese oxide precursors, respectively. DCH18C6 (C_20_H_36_O_6_, >98.0%, Tokyo Chemical Industry Co., Ltd., Japan) was used as the main chemical agent for preparing purple benzene. Manganese(IV) oxide powder (85% purity, Kanto Chemical Co., Inc., Japan) was used as a commercial MnO_2_ sample for comparing the dye removal performance with the as-prepared adsorbents. Ethanol (C_2_H_5_OH, >99.5%), hydrochloric acid (HCl, 35–37%), and a sodium hydroxide solution (NaOH, 48–50%) were purchased from Kanto Chemical Co., Inc., Japan. MB (C_16_H_18_ClN_3_S·3H_2_O, 98.5% purity, Kishida Chemical Co., Ltd., Japan), Rh B (C_28_H_31_ClN_2_O_3_, Guaranteed reagent, Wako Pure Chemical Industries Ltd., Japan) and MO (C_14_H_14_N_3_NaO_3_S, ACS reagent, dye content 85%, Sigma-Aldrich, Japan) were selected as typical cationic and anionic organic dyes. Distilled water was obtained from an Aquarius water distillation apparatus (RFD250NB, Advantec, Japan) with a resistivity of 18.2 MΩ cm at 25 °C. All chemicals were used without further purification.

### Synthesis of the MnO_2_-carbon hybrid by SPP

**Figure 9:**
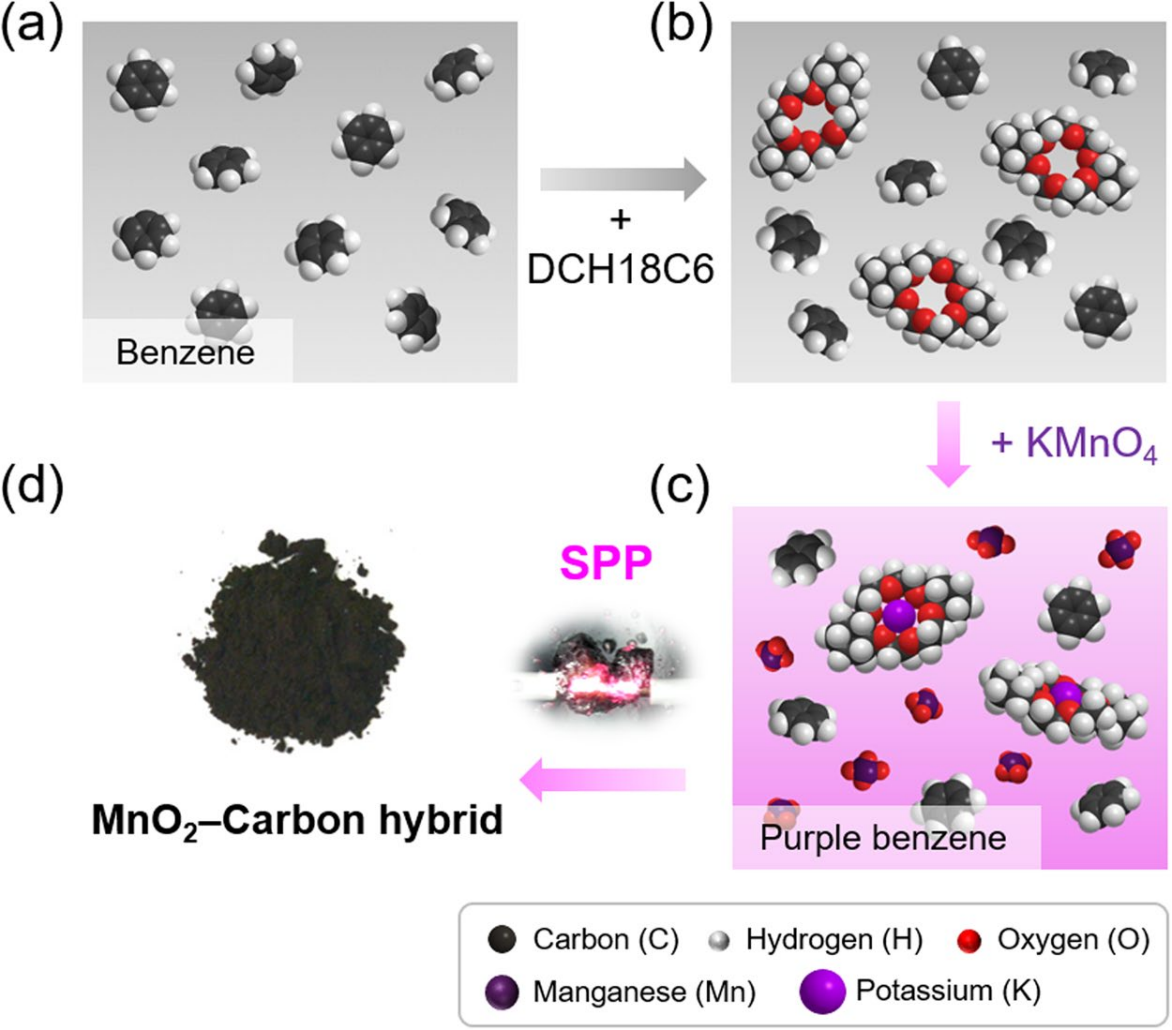
Schematic for the synthesis of the MnO_2_-carbon hybrid from purple benzene by SPP: (**a**) benzene solvent, (**b**) dicyclohexano-18-crown-6 ether-added benzene, (**c**) purple benzene, and (d) MnO_2_-carbon hybrids synthesized from purple benzene by SPP.

The MnO_2_-carbon hybrid was successfully synthesized by a one-pot SPP in purple benzene derived from the K^+^ (DCH18C6) complex. Purple benzene was prepared as follows (Fig. 9). DCH18C6 (50 mM) was added to benzene (100 mL). After vigorous stirring for a few minutes, KMnO_4_ (5 mM) was added and sufficiently mixed until a homogeneous purple solution was obtained without the remaining solid KMnO_4_. As-prepared purple benzene was transferred into a glass reactor with a volume of 100 mL, consisting of a pair of tungsten electrodes (Ø 1 mm, 99.5% purity, Nilaco, Japan) insulated with ceramic tubes and connected to a bipolar pulse power supply (Kurita, Japan) for generating plasma in the liquid-phase precursor. The plasma discharging conditions for frequency, pulse width, voltage, and gap distance of the metal electrode were maintained constant at 25 kHz, 0.8 μs, 1.4–1.6 kV, and 1 mm, respectively. Figure S5^†^ shows the photograph of the purple benzene precursor and the schematic of the SP apparatus in detail. SP was allowed to proceed at ambient temperature and pressure for 10 min under constant stirring during operation to ensure a homogeneous chemical reaction. The resulting MnO_2_-carbon hybrid obtained from SPP was collected by vacuum filtration and rinsed several times with ethanol, followed by air drying in an oven at 85 °C for 12 h. The production rate of MnO_2_-carbon hybrid was approx. 20 mg min^–1^ in the purple benzene during discharging. In addition, to demonstrate the effect of the MnO_2_-carbon hybrid, reference samples were prepared from pure benzene and benzene with DCH18C6 ethers without KMnO_4_ under the same SP conditions as stated above. The resulting products obtained using pure benzene, benzene with crown ether (DCH18C6), and purple benzene precursors were designated as C-Bz (carbon synthesized from benzene), C-CE-Bz (carbon synthesized from crown ether-benzene), and MnO_2_-C-PBz (MnO_2_-carbon hybrid synthesized from purple benzene), respectively.

### Material characterization

The fabricated samples were characterized by X-ray diffraction (XRD, SmartLab, Rigaku Co., Ltd., Japan) with Cu Kα (λ = 1.5418 Å) radiation operating at 45 kV and 200 mA and by Raman spectroscopy (inVia Raman Microscope, Renishaw Co. Ltd., UK) with a solid-state laser operating at 532 nm to examine the structural features. N_2_ adsorption/desorption isotherms were recorded on a BELSORP mini II analyzer (MicrotracBEL Corp.) at 77 K in liquid nitrogen to characterize the surface area and pore structure of the adsorbents. The specific surface area (SSA) was determined by the Brunauer–Emmett–Teller (BET) method, and the pore size distribution was calculated from the Barrett–Joyner–Halenda analysis. Morphologies were observed by field-emission scanning electron microscopy (FE-SEM, S-4800, HITACHI High Technologies Co., Ltd., Japan) at an accelerating voltage of 10 kV and by transmission electron microscopy (TEM, JEM-2500SE, JEOL, Japan) at an accelerating voltage of 200 kV. Energy-dispersive X-ray spectroscopy (EDS) and elemental mapping images were obtained on an FE-SEM system equipped with an EDS system (EMAX Energy, Horiba Ltd., Japan). X-ray photoelectron spectroscopy (XPS) was carried out on a PHI 5000 VersaProbe II (ULVAC-PHI, Inc., Japan) with Mg Kα radiation to examine the surface chemistry of materials.

### Adsorption studies

Adsorption capacities for MB from aqueous solutions were analyzed by batch experiments. To observe the adsorption of anionic and cationic organic dyes on the as-prepared samples, MO, MB and Rh B were selected. Adsorption kinetic experiments were carried out under different conditions of initial dye concentration (10, 25, 50, 100, 200, and 300 mg L^−1^), solution pH (2, 4, 6.5, 8, 10, and 12), and contact time (0–240 min). The pH was adjusted using a 0.1 M HCl or 0.1 M NaOH solution, and adsorption experiments were carried out at room temperature (297 K) and under natural pH. The adsorbent dose was maintained constant at 0.6 g L^−1^, and adsorption experiments were carried out in a 150 mL conical flask with 60 mg of the adsorbent and 100 mL of the dye stock solution. During the tests, the conical flasks were wrapped in aluminum foil to intercept surrounding light so as to minimize other effects. UV-visible spectroscopy (Shimadzu UV-3600, Japan) was utilized to determine dye removal efficiency and adsorption capacity. The solution was filtered using a 0.45 μm membrane filter (PTFE, ADVANTEC, Japan) before characterization. The dye removal efficiency (*R*) and adsorption capacity at equilibrium (*q*_*e*_) were evaluated from the UV-visible spectra using the following equations:

8 $$R\,({\rm{ \% }})=\frac{({C}_{0}\,-\,{C}_{t})}{{C}_{0}}\,\times \,100$$

9 $${q}_{e}=\frac{({C}_{0}\,-\,{C}_{e})}{W}\,\times \,V$$

where C_0_, C_t_, and C_e_ represent initial dye concentration, dye concentration at contact time (*t*), and dye concentration at equilibrium (mg L^−1^), respectively. *W* and *V* represent adsorbent dosage (mg) and dye solution volume (L), respectively.

### Reusability tests

Reusability tests were carried out to evaluate the stability and reusability of adsorbents. A total of 60 mg of adsorbents was added in 100 mL of an MB solution with a C_0_ of 100 mg L^−1^ at natural pH (6.5 ± 0.5) for 180 min. After the adsorption of MB on the adsorbent, the solution phase was obtained by filtration using a 0.45 μm membrane filter, and the solid phase (adsorbent) was collected by centrifugation, followed by washing two times using ethanol with 0.1 M NaOH (1 wt%). The washed adsorbent was dried at 65 °C for 2 hrs, and recyclability tests were carried out seven times under the same conditions and procedures as stated above.

## Electronic supplementary material

Supplementary Information
