# Supplementary material for: One-pot synthesis of purple benzene-derived MnO2-carbon hybrids and synergistic enhancement for the removal of cationic dyes
Source: Sci Rep. 2018 Mar 12;8:4342. doi: 10.1038/s41598-018-22203-1 (PMC5847607; doi:10.1038/s41598-018-22203-1)
Supplement: Supplementary file 1 — Supplementary Information [file 41598_2018_22203_MOESM1_ESM.docx]

Supplementary Information

**One-pot synthesis of purple benzene-derived MnO_2_–carbon hybrids and synergistic enhancement for the removal of cationic dyes**

Hyemin Kim^a^ and Nagahiro Saito^a,b*^

**Author affiliations:**

^a^Department of Materials Science and Engineering, Graduate School of Engineering,

Nagoya University, Furo-cho, Chikusa-ku, Nagoya 464-8603, Japan

^b^CREST-JST, Nagoya University, Furo-cho, Chikusa-ku, Nagoya 464-8603, Japan

***Corresponding author**:

hiro@rd.numse.nagoya-u.ac.jp

Phone/Fax: +81-52-789-3259

**XRD patterns**


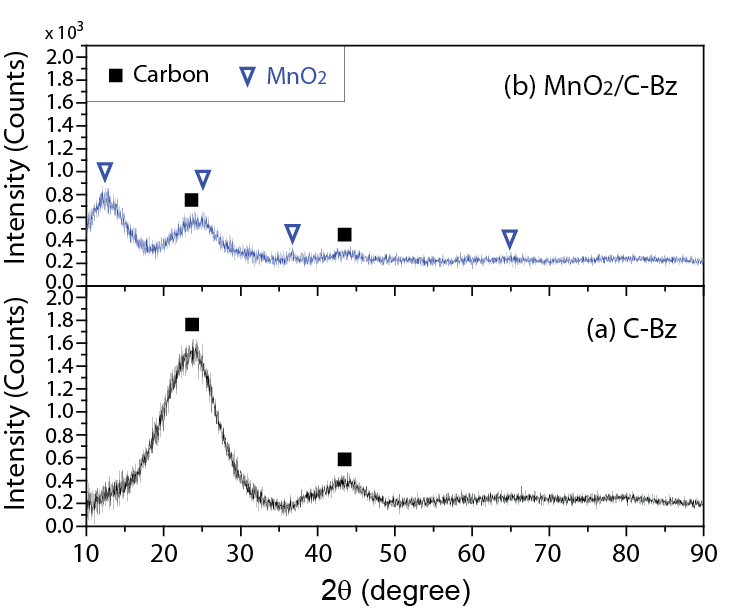


Figure S1 Comparison of XRD patterns; (a) C-Bz and (b) MnO_2_/C-Bz prepared by mixing of C-Bz in KMnO_4_ aqueous solution.

To verify the distribution of MnO_2_ in the carbon matrix of MnO_2_-C-PBz, the MnO_2_ was deposited on the carbon surface (i.e. MnO_2_/C-Bz) by 2-step synthesis method for comparing before/after deposition. The MnO_2_/C-Bz sample was prepared by dispersing the C-Bz in KMnO_4_ aqueous solution and sonication treatment. As can be seen in Fig. S1, similar to the XRD results of MnO_2_-C-PBz (Fig. 1(a)), the intensity of (002) plane of carbon (■) was significantly reduced and new peaks (▽) were revealed at around 12.6°, 24.8°, 36.5° and 65.5°, corresponding to MnO_2_ (JCPDS #80-1098). Therefore, there is a possibility that MnO_2_ is formed and dispersed on the carbon surface for the MnO_2_-C-PBz. This can be deduced from the SAED pattern and EDS mapping results in Fig. 3.

**MnO_2_ synthesized by solution plasma (MnO_2_-SP)**

In our previous study, porous MnO_2_ was synthesized by the solution plasma process^28^. To confirm the synergistic effects of the MnO_2_–carbon hybrids for dye removal and stability, the carbon-free MnO_2_ sample (MnO_2_-SP) was used. Table S1^†^ summarizes the properties of MnO_2_-SP.

Table S1 Structural and physical properties of MnO_2_ (MnO_2_-SP) synthesized by the solution plasma process_._

| **(a) X-ray diffraction pattern** | |
| --- | --- |
| 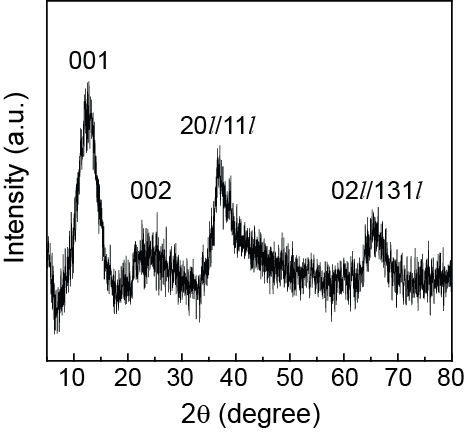 | ‣ Potassium birnessite-type MnO_2_ (*δ*-MnO_2_)  (prismatic crystal and *C_2/m_* space group) |
| **(b) Raman spectrum** | |
| 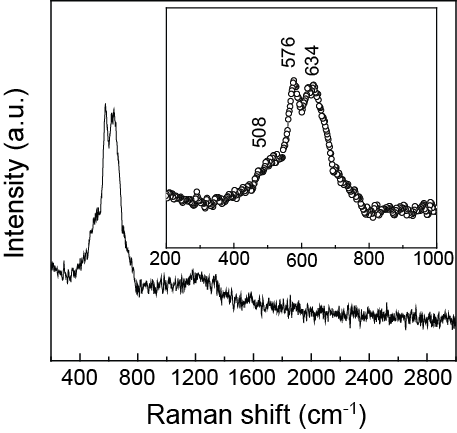 | ‣ 630–645 (cm^−1^): symmetric stretching vibration of the [Mn–O] bond in the MnO_6_ octahedra  ‣ 570–580 (cm^−1^): stretching vibration mode  of the [Mn–O] bond in the MnO_6_ octahedral basal plane |
| **(c) Physical properties** (N_2_ adsorption/desorption isotherms and pore size distribution (inset)) | |
| 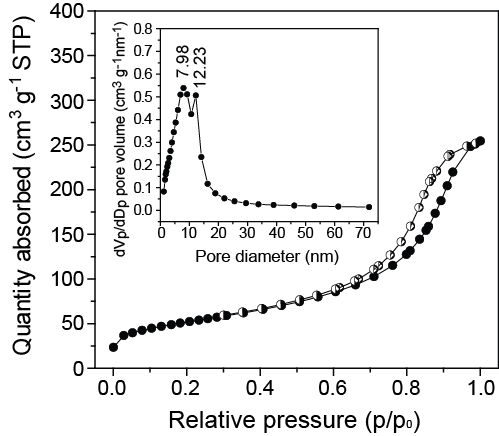 | ‣ BET specific surface area: 169.1 m^2^ g^−1^  ‣ Total pore volume: 9.3 cm^3^ g^−1^  ‣ Mean pore size: 0.39 nm |

**Dye removal test of MnO_2_-carbon hybrid (Rhodamine B: cationic dye, MO: anionic dye)**


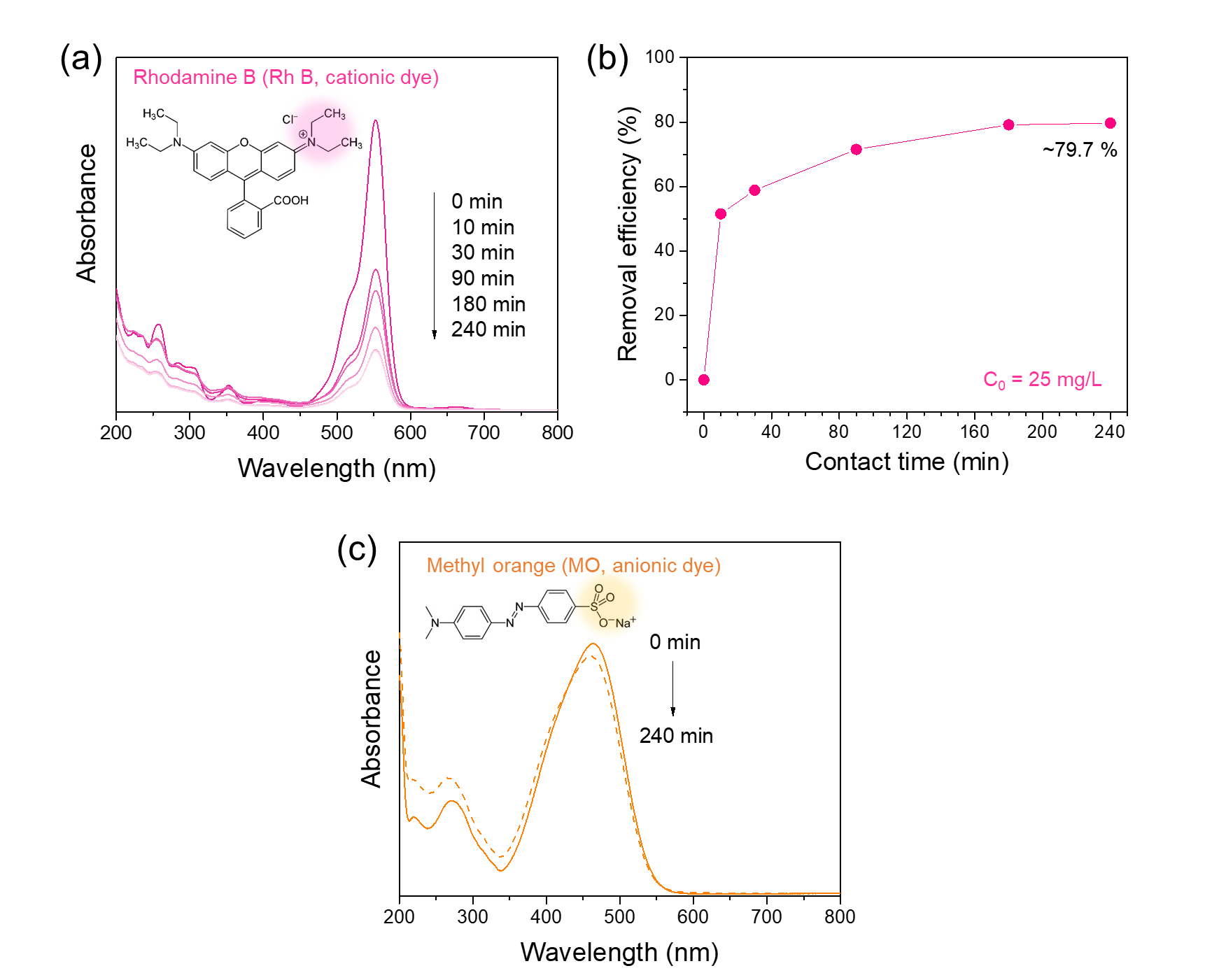


Fig. S2 (a) UV–visible adsorption spectra of Rh B dye solution as a function of contact time, (b) dependence of time on Rh B removal efficiency (C_0_ = 25 mg L^–1^) and (c) UV–visible adsorption spectra of MO dye solution before after adsorption on MnO_2_-C-PBz (297 K, 0.6 g L^−1^ of adsorbent, and natural pH).

As shown in Fig. S2, MnO_2_–carbon hybrid sample showed favorable adsorption behavior for Rh B (Fig. S2 (a and b)) while the adsorption is negligible for MO (Fig. S2 (c)). This results exhibits that as-prepared MnO_2_–carbon hybrid is effective adsorbent for cationic dyes.


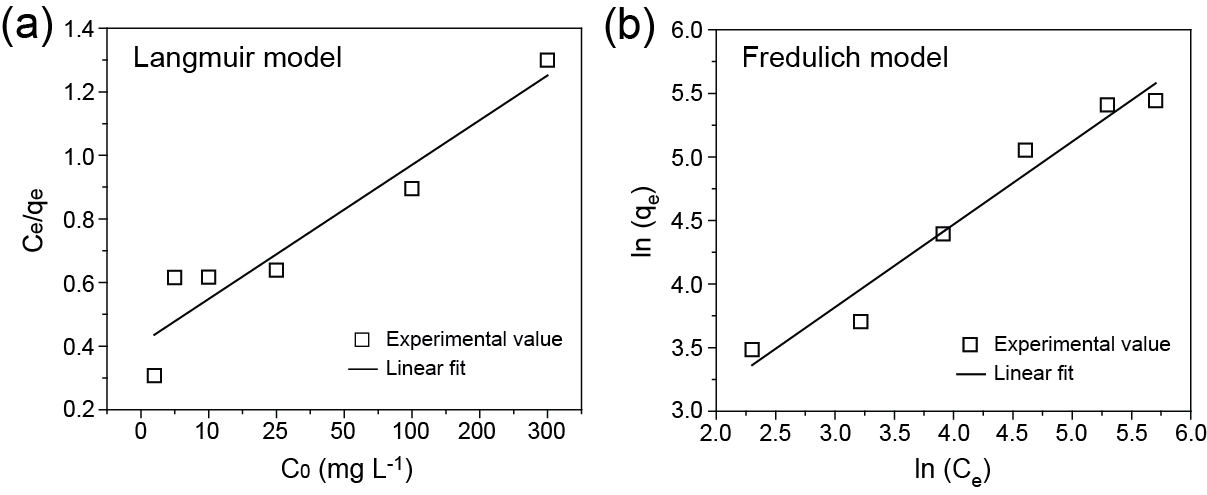


Figure S3 (a) Langmuir and (b) Freundlich adsorption isotherms models for the adsorption of MB on MnO_2_-C-PBz (C_0_ = 25, 50, and 100 mg L^−1^, adsorbent = 0.6 g L^−1^, contact time = 240 min).

Table S2 Comparison of the maximum adsorption capacities of related materials reported in the literatures including carbon, metal oxide, and metal oxide–carbon hybrid adsorbents for methylene blue.

| Type | Adsorbents | Maximum adsorption capacity, *q_max_* (mg g^−1^) | Temp. | pH | Ref. |
| --- | --- | --- | --- | --- | --- |
| Carbon-based | Activated carbon (AC) | 345 | 293 K | 7 | [S1] |
|  | 3D hierarchically porous carbon (3D HPC) | 574.42 | 298 K | Natural | [S2] |
|  | Functionalized CNT (f-CNT) | 99.80 ± 2.78 | 298.15 K | Natural | [S3] |
|  | Graphene oxide (GO) | 144.92 | 298 K | 5.4 | [S4] |
| Metal oxide | Mesoporous birnessite (δ-MnO_2_) | 90.83 | 293 K | 7 | [S5] |
|  | TiO_2_/SiO_2_/Fe_3_O_4_ hollow  magnetic microspheres **(**TSF-HMMS**)** | 147.0 | 298 K | 6.82 | [S6] |
|  | Biomorphic SiO_2_ materials (HS005) | 74.02 | 298 K | 11 | [S7] |
|  | Magnetic iron oxide nanopowder (MnP) | 25.54 | 298 K | 6.5 | [S8] |
| Metal oxide-carbon hybrids | Zeolite–activated carbon (Z–AC) | 143.47 | 303 K | - | [S9] |
|  | Graphene–Fe_3_O_4_ with carbon (GFC) | 73.26 | 298 K | - | [S10] |
|  | ZnO nanorod–activated carbon  (ZnO–NRs–AC) | 89.29 | Room temp. | 6 | [S11] |
|  | Activated carbon–MnO_2_ nanocomposite  (AC-MnO_2_-NC) | 45.454 | - | - | [S12] |
|  | MnO_2_ nanoparticle-loaded activated carbon (MnO_2_-NP-AC) | 234.20 | Room temp. | 7 | [S13] |
|  | Graphene nanosheet/magnetite (GNS/Fe_3_O_4_) | 43.82 | 298 K | - | [S14] |
|  | Mesoporous zeolite–Aactivated carbon  (Z-AC) | 285.71 | 323 K | 7 | [S15] |
|  | MnO_2_–carbon hybrid (MnO_2_-C-PBz) | 357.14 | 297 K | 6.5 ± 0.5 | This work |


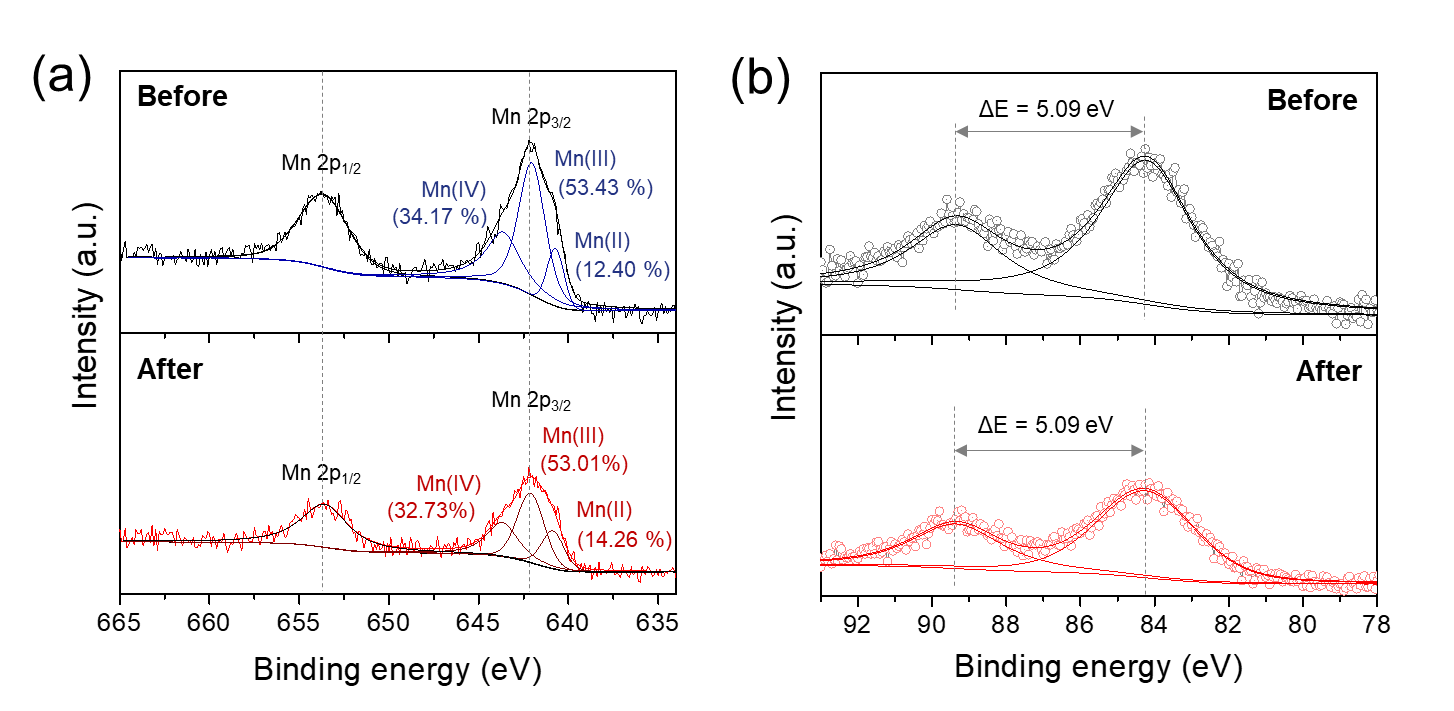


**Figure S4** The XPS analysis of the initial (before) and residual solid (after) of MnO_2_-C-PBz after MB adsorption test; (a) Mn 2p and (b) Mn 3s.

**Synthesis of the MnO_2_–carbon hybrid by the solution plasma process**


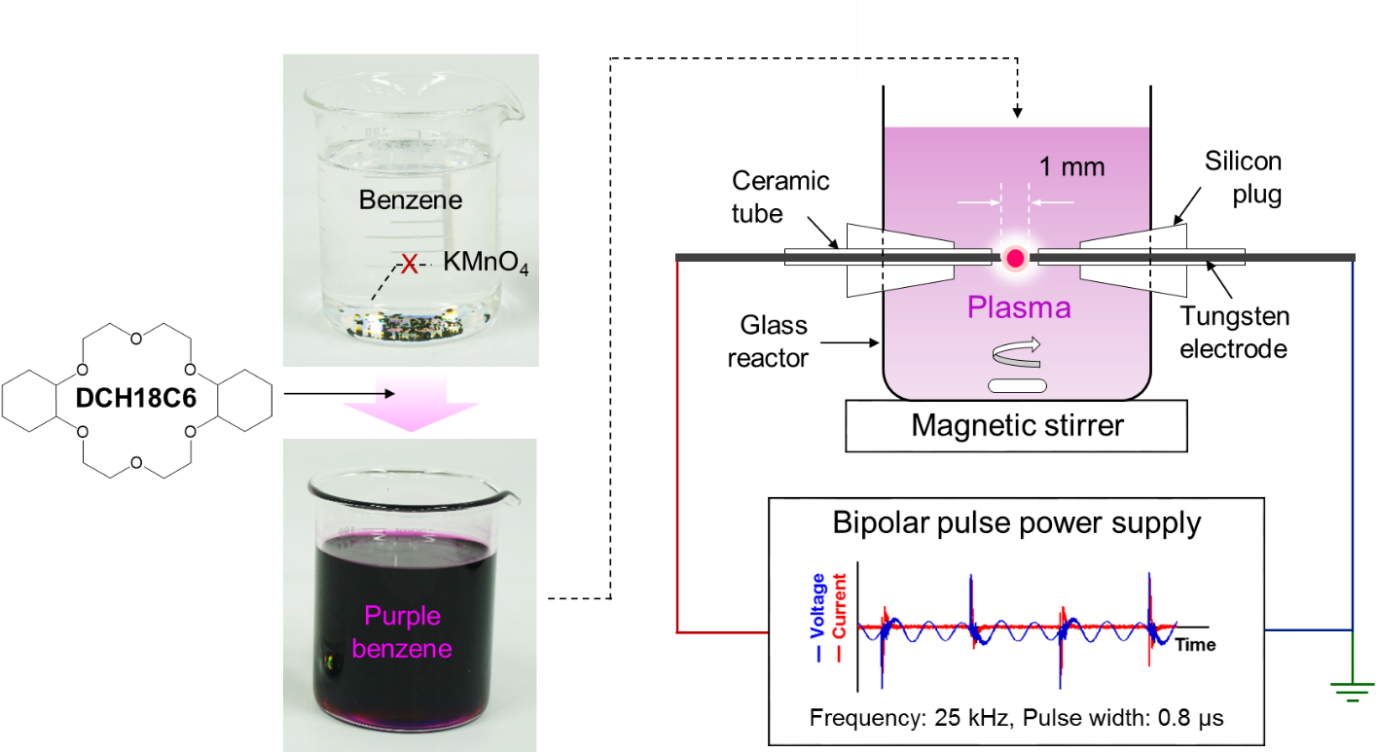


**Figure S5** Photograph of purple benzene derived from the K^+^(DCH18C6) complex and experimental set-up for the solution plasma process.

**References related to the maximum monolayer adsorption capacities**

1. El Qada, E. N., Allen, S. J. & Walker, G. M. Adsorption of methylene blue onto activated carbon produced from steam activated bituminous coal: a study of equilibrium adsorption isotherm. *Chem. Eng. J.* **124**, 103-110 (2006).
2. Dai, J. *et al.* Designed preparation of 3D hierarchically porous carbon material via solvothermal route and in situ activation for ultrahigh-efficiency dye removal: adsorption isotherm, kinetics and thermodynamics characteristics. *RSC Adv.* 6, 3446-3457 (2016).
3. Ortega, P. F. *et al.* Thermodynamic Study of Methylene Blue Adsorption on Carbon Nanotubes Using Isothermal Titration Calorimetry: A Simple and Rigorous Approach. *J. Chem. Eng. Data.,* 62, 729-737 (2017).
4. Li, Y. *et al.* Methylene blue adsorption on graphene oxide/calcium alginate composites. *Carbohydr. Polym.* 95, 501-507 (2013).
5. Pang, J. et al. Adsorption behaviors of methylene blue from aqueous solution on mesoporous birnessite. *J. Taiwan. Inst. Chem. Eng.* (2017).
6. Zhang, H., Li, X., He, G., Zhan, J. & Liu, D. Preparation of magnetic composite hollow microsphere and its adsorption capacity for basic dyes. *Ind. Eng. Chem. Res.* 52, 16902-16910 (2013).
7. Cui, R. *et al.* Two-Dimensional Porous SiO_2_ Nanostructures Derived from Renewable Petal Cells with Enhanced Adsorption Efficiency for Removal of Hazardous Dye. *ACS Sustain. Chem. Eng.* 5, 3478-3487 (2017).
8. Păcurariu, C., Paşka, O., Ianoş, R. & Muntean, S. G. Effective removal of methylene blue from aqueous solution using a new magnetic iron oxide nanosorbent prepared by combustion synthesis. *Clean Technol. Environ. Policy.* 18, 705-715 (2016).
9. Khanday, W., Marrakchi, F., Asif, M. & Hameed, B. Mesoporous zeolite–activated carbon composite from oil palm ash as an effective adsorbent for methylene blue. *J. Taiwan. Inst. Chem. Eng.* 70, 32-41 (2017).
10. Fan, W. *et al.* Hybridization of graphene sheets and carbon-coated Fe_3_O_4_ nanoparticles as a synergistic adsorbent of organic dyes. *J. Mater. Chem.* 22, 25108-25115 (2012).
11. Dil, E. A. *et al.* Modeling of quaternary dyes adsorption onto ZnO–NR–AC artificial neural network: analysis by derivative spectrophotometry. *J. Ind. Eng. Chem.* 34, 186-197 (2016).
12. Santhi, M. & Kumar, P. Adsorption of basic dye, methylene blue by a novel activated carbon prepared from typha angustata (L.). *Chem. Sci. Trans.* 4, 389-400 (2015).
13. Asfaram, A., Ghaedi, M., Hajati, S. & Goudarzi, A. Ternary dye adsorption onto MnO_2_ nanoparticle-loaded activated carbon: derivative spectrophotometry and modeling. *RSC Adv.* 5, 72300-72320 (2015).
14. Ai, L., Zhang, C. & Chen, Z. Removal of methylene blue from aqueous solution by a solvothermal-synthesized graphene/magnetite composite. *J. Hazard. Mater*. 192, 1515-1524 (2011).
15. Khanday, W., Marrakchi, F., Asif, M. & Hameed, B. Mesoporous zeolite–activated carbon composite from oil palm ash as an effective adsorbent for methylene blue. *J. Taiwan Inst. Chem. Eng.* 70, 32-41 (2017).
